# High Serum Adipocyte Fatty Acid Binding Protein Is Associated with Metabolic Syndrome in Patients with Type 2 Diabetes

**DOI:** 10.1155/2016/8380279

**Published:** 2016-11-29

**Authors:** Jer-Chuan Li, Du-An Wu, Jia-Sian Hou, Yi-Maun Subeq, Hsin-Dean Chen, Bang-Gee Hsu

**Affiliations:** ^1^Division of Metabolism and Endocrinology, Buddhist Tzu Chi General Hospital, Hualien, Taiwan; ^2^School of Medicine, Tzu Chi University, Hualien, Taiwan; ^3^Division of Nephrology, Buddhist Tzu Chi General Hospital, Hualien, Taiwan; ^4^Institute of Medical Sciences, Tzu Chi University, Hualien, Taiwan; ^5^Department of Nursing, Tzu Chi University, Hualien, Taiwan

## Abstract

Adipocyte fatty acid binding protein (A-FABP) is a key mediator of obesity-related metabolic syndrome (MetS). The aim of this study was to evaluate the relationship between A-FABP concentration and MetS in type 2 diabetes mellitus (DM) patients. Fasting blood samples were obtained from 165 type 2 DM volunteers. MetS and its components were defined using diagnostic criteria from the International Diabetes Federation. Among 165 DM patients, 113 patients (68.5%) had MetS. Diabetic persons who had MetS had significantly higher A-FABP levels (*P* < 0.001) than those without MetS. Female DM persons had higher A-FABP level than man (*P* < 0.001). No statistically significant differences in A-FABP levels were found in use of statin, fibrate, or antidiabetic drugs. Multivariate forward stepwise linear regression analysis revealed that body fat mass (*P* < 0.001), logarithmically transformed creatinine (log-creatinine; *P* < 0.001), female DM patients (*P* < 0.001), and logarithmically transformed high sensitive C-reactive protein (log-hs-CRP; *P* = 0.013) were positively correlated, while albumin (*P* = 0.004) and glomerular filtration rate (GFR; *P* = 0.043) were negatively correlated with serum A-FABP levels in type 2 DM patients. In this study, higher serum A-FABP level was positively associated with MetS in type 2 DM patients.

## 1. Introduction

Metabolic syndrome (MetS) is a clustering of metabolic risk factors for atherosclerotic cardiovascular disease and diabetes mellitus (DM) [1]. There are more than 415 million people affected by DM worldwide and it is one of the most important public health challenges of the twenty-first century [2].

Adipocyte fatty acid binding protein (A-FABP) is one of the most abundant proteins in mature adipocytes, which is known for the ability to bind fatty acids and related compounds throughout various cellular compartments, including the peroxisomes, mitochondria, endoplasmic reticulum, lipid droplets, and nucleus [3, 4]. A-FABP has been shown to affect insulin sensitivity, lipid metabolism, and lipolysis in animal studies [3]. Furthermore, studies also found that A-FABP is a key mediator for the obesity-related cardiovascular disease and is positively associated with MetS [3, 5]. Our previous studies noted that A-FABP was associated with MetS in coronary artery disease and in hemodialysis patients [6, 7]. The aim of this study was to investigate the relationship between fasting serum A-FABP level and the MetS among type 2 DM patients.

## 2. Materials and Methods

### 2.1. Patients

There were 165 type 2 DM patients enrolled from a medical center in Hualien, Taiwan, from November 2014 through March 2015. The Protection of the Human Subjects Institutional Review Board of Tzu Chi University and Hospital approved this study. All patients provided their informed consents before participating in this study. Blood pressure (BP) was measured by trained staff in the morning using standard mercury sphygmomanometers with appropriate cuff sizes after sitting for at least 10 minutes. Systolic BP (SBP) and diastolic BP (DBP) were taken 3 times at 5 min intervals and were averaged for analysis. Patients who were diagnosed with hypertension were defined as SBP ≥ 140 mmHg and/or DBP ≥ 90 mmHg or have received any antihypertensive medication in the past 2 weeks. Patients were excluded if they had an acute infection, acute myocardial infarction, heart failure, and malignancy at the time of blood sampling, or if they refused to provide informed consent for the study.

### 2.2. Anthropometric Analysis

Body weight of participant was measured in light clothing and without shoes to the nearest 0.5 kilograms, and body height was measured to the nearest 0.5 cm. Waist circumference was measured using a tape measurement around the waist from the point between the lowest ribs and the hip bones with the hands on the hips. Body mass index (BMI) was calculated as the weight in kilograms divided by the height in meters squared. Bioimpedance measurements of fat mass were performed at the bedside according to the standard tetrapolar whole body (hand-foot) technique, using a single-frequency (50 kHz) analyzer (Biodynamic-450, Biodynamics Corporation, Seattle, USA). Measurements were carried out by the same operator [6–8].

### 2.3. Biochemical Investigations

Fasting blood samples (approximately 5 mL) of all participants were immediately centrifuged at 3000 ×g for 10 min. Serum levels of albumin, blood urea nitrogen (BUN), creatinine, fasting glucose, glycated hemoglobin (HbA1c), total cholesterol (TCH), triglycerides (TG), high-density lipoprotein cholesterol (HDL-C), low-density lipoprotein cholesterol (LDL-C), and high-sensitivity C-reactive protein (hs-CRP) were measured using an autoanalyzer (Siemens Advia 1800, Siemens Healthcare GmbH, Henkestr, Germany) [6–8]. Serum A-FABP levels were measured using a commercially available enzyme immunoassay (EIA; SPI-BIO, Montigny le Bretonneux, France) [6–8]. Serum insulin levels were measured using the commercially available enzyme-linked immunosorbent assay (ELISA) (Labor Diagnostika Nord, Nordhorn, Germany) [9, 10]. Insulin resistance was evaluated using a homeostasis model assessment of insulin resistance (HOMA-IR) as follows: HOMA-IR = fasting plasma glucose (mg/dL) × fasting serum insulin (*μ*U/mL)/405 [9, 10]. The estimate glomerular filtration rate (GFR) was calculated using the Modification of Diet in Renal Disease (MDRD) study equation in this study.

### 2.4. Metabolic Syndrome and Its Components

The prevalence of MetS was defined using the International Diabetes Federation definition [11]. People were classified as having MetS if they had central (abdominal) obesity with a waist circumference ≥ 90 cm (men) or ≥80 cm (women) (Chinese criteria) and matched two or more of the following criteria: fasting serum glucose ≥ 110 mg/dL, triglycerides ≥ 150 mg/dL, HDL-C level < 40 mg/dL in men or <50 mg/dL in women, or blood pressure ≥ 130/85 mmHg. The use of antihypertensive drug was considered as indicative of high blood pressure in this analysis. Type 2 DM was determined according to World Health Organization criteria [12]. A patient was considered DM patient if the fasting plasma glucose was ≥126 mg/dL, or if antidiabetic therapy was used [6–8].

### 2.5. Statistical Analysis

Data were tested for normal distribution using the Kolmogorov-Smirnov test. Normally distributed data are expressed as the mean ± standard deviation (SD) and comparisons between patients were performed using Student's independent *t*-test (two-tailed). Data are not normally distributed are expressed as medians and interquartile ranges and comparisons between patients were performed using the Mann–Whitney *U* test (age, TG, fasting glucose, HbA1c, BUN, creatinine, hs-CRP, insulin, and HOMA-IR). Data expressed as the number of patients were analyzed by *χ* ^2^ test, because age, TG, fasting glucose, HbA1c, BUN, creatinine, hs-CRP, insulin, and HOMA-IR were not normally distributed and underwent base 10 logarithmic transformations to achieve normality. Clinical variables that correlated with serum A-FABP levels in DM patients were evaluated using univariate linear regression analysis. Variables that were significantly associated with A-FABP levels in DM patients were tested for independency in multivariate forward stepwise regression analysis. Data were analyzed using SPSS for Windows (version 19.0; SPSS Inc., Chicago, IL, USA). A *P* value < 0.05 was considered statistically significant.

## 3. Results

Clinical characteristics of the 165 type 2 DM patients are presented in Table 1. One hundred thirteen DM persons (68.5%) had MetS, and 52 DM persons (31.5%) did not. Diabetic persons who had MetS had significantly higher serum fasting A-FABP levels than those without MetS (*P* < 0.001). Compared with DM patients without MetS, those with MetS showed a much higher proportion of females (*P* = 0.002) and as expected more hypertension (*P* = 0.001), higher waist circumference (*P* < 0.001), body weight (*P* < 0.001), and BMI (*P* < 0.001), higher fasting glucoses (*P* = 0.033), TG (*P* = 0.001), and lower HDL-C concentrations (*P* < 0.001) since MetS represents a constellation of hypertension, abdominal obesity, impaired fasting glucose, and dyslipidemia. Moreover, DM patients with MetS had higher body fat mass (*P* < 0.001), HbA1c level (*P* = 0.007), BUN (*P* = 0.020), hs-CRP (*P* < 0.001), insulin level (*P* < 0.001), HOMA-IR (*P* < 0.001), and lower GFR (*P* = 0.021).

Clinical characteristics and serum A-FABP values for the 165 DM patients are presented in Table 2. A-FABP level was statistically significantly higher in female DM persons than male DM persons (*P* < 0.001). No statistically significant differences in A-FABP levels were found as a function of smoking; presence of hypertension; or use of statin, fibrate, or antidiabetic drugs.

Univariate linear analysis of clinical variables associated with fasting serum A-FABP levels in DM patients is presented in Table 3. Height (*r* = −0.293; *P* < 0.001) and serum albumin level (*r* = −0.259; *P* = 0.001) were negatively correlated, while BMI (*r* = 0.173; *P* = 0.026), body fat mass (*r* = 0.394; *P* < 0.001), SBP (*r* = 0.324; *P* < 0.001), DBP (*r* = 0.173; *P* = 0.026), TCH (*r* = 0.179; *P* = 0.022), and logarithmically transformed TG (log-TG, *r* = 0.227; *P* = 0.003) were positively correlated with serum A-FABP levels in type 2 DM patients. Furthermore, log-HbA1c (*r* = 0.225; *P* = 0.004), log-BUN (*r* = 0.363; *P* < 0.001), log-creatinine (*r* = 0.257; *P* = 0.001), log-hs-CRP (*r* = 0.212; *P* = 0.006), log-insulin (*r* = 0.214; *P* = 0.006), and log-HOMA-IR (*r* = 0.218; *P* = 0.005) were positively correlated, while GFR (*r* = −0.343; *P* < 0.001) was negatively correlated with serum A-FABP levels in this study.

Multivariate forward stepwise linear regression analysis of the variables significantly associated with fasting serum A-FABP levels revealed that body fat mass (adjusted *R* ^2^ change = 0.150; *P* < 0.001), log-creatinine (adjusted *R* ^2^ change = 0.140; *P* < 0.001), female DM patients (adjusted *R* ^2^ change = 0.051; *P* < 0.001), serum albumin level (adjusted *R* ^2^ change = 0.029; *P* = 0.004), log-hs-CRP (adjusted *R* ^2^ change = 0.020; *P* = 0.013), and GFR (adjusted *R* ^2^ change = 0.012; *P* = 0.043) were independent predictors of these values for type 2 DM patients (Table 4).

## 4. Discussion

Our study showed that fasting A-FABP levels were higher in type 2 DM patents with MetS, and body fat mass, creatinine, female, albumin, hs-CRP, and GFR were independent predictors of serum A-FABP levels in type 2 DM patients.

MetS is defined by the presence of central obesity, hyperglycemia, type 2 DM, hypertension, and dyslipidemia [11]. The prevalence of MetS was 46.9% for males and 65.1% for females in type 2 DM patients in a Korean study [13] and 78.6% in type 2 DM patients in Brazil [14]. In this study, the prevalence of MetS in type 2 DM patients is 68.5%. MetS is strongly associated with insulin resistance and inflammation [11]. Patients with type 2 DM and MetS showed significantly higher blood CRP and insulin resistance than type 2 DM without MetS [13]. Our study noted that diabetic persons who had MetS had significantly higher in-body fat mass, HbA1c level, hs-CRP, insulin level, and HOMA-IR than those diabetic persons without MetS. Females are at greater risk of obesity and central adiposity due to their increased propensity to gain fat [15]. Our results found that diabetic persons who had MetS had higher trend in female gender. MetS and its components are associated with the development of chronic kidney disease and microalbuminuria or overt proteinuria [16]. Type 2 DM patients with MetS are associated with the reduction of GFR [14]. Our study also noted that MetS in patients with type 2 DM had higher serum BUN level and lower GFR.

Studies in A-FABP knockout mice and A-FABP inhibitor treated animals suggest that A-FABP has an important role in lipolysis and regulating insulin sensitivity [3, 5, 17, 18]. Recent studies also demonstrated A-FABP expression in macrophages and modulate inflammatory responses and cholesterol ester accumulation [3, 5, 19]. All of these results indicate that A-FABP has an important role in the development of major components of the MetS through its distinct actions in adipocytes and macrophages. Serum A-FABP levels predict the development of the MetS in a Chinese and a Korean cohort study [20, 21] and plasma A-FABP level was found to be a strong predictor of type 2 DM independently of the traditional risk factors including obesity, insulin resistance, or glycemic indexes [22]. Cabré et al. noted A-FABP levels positively correlated to the MetS in type 2 DM subjects [23]. Our study also noted higher serum A-FABP level in type 2 DM patients who had MetS. Hao et al. noted serum A-FABP levels significantly higher in women than men [24]. Our study also noted that female diabetic patients had higher A-FABP levels than man. In type 2 DM patients, A-FABP levels were positively correlated with BMI, TG, TCH, SBP, waist circumference, IL-6, TNF-*α*, and hs-CRP [23, 25]. Furthermore, A-FABP levels were positively correlated with plasma TG, apolipoprotein C-III, and all the components of TG-rich lipoproteins in type 2 diabetic subjects [26]. A-FABP levels positively correlated with BMI, glucose, insulin levels, HOMA-IR, and CRP in morbidly obese women [27]. Subjects in the highest A-FABP tertile at baseline exhibited higher values for BMI, body fat mass, blood pressure, fasting glucose, TCH, TG, LDL-C, insulin, and HOMA-IR in a total of 465 participants in a Korean study [21]. A-FABP was independently associated with body fat mass in a cohort of Chinese women without DM [28]. Serum A-FABP concentrations were positively associated with serum creatinine and albumin and predict a worse hospital outcome in critically ill patients with sepsis [29]. Since serum creatinine is a component of the GFR estimation. Studies also noted serum A-FABP4 levels correlated positively with serum creatinine and negatively with GFR in type 2 DM patients [30, 31]. Serum A-FABP level was independently associated with macrovascular complications in type 2 DM patients [31]. We also noted that BMI, body fat mass, SBP, DBP, TCH, log-TG, log-creatinine, log-HbA1c, log-hs-CRP, log-insulin, and log-HOMA-IR were positively correlated with A-FABP levels, while height, albumin, and GFR were negatively correlated with A-FABP levels in our DM subjects. After adjustment for a variety of confounders in multivariable forward stepwise linear regression analysis, body fat mass, log-creatinine, female DM patients, and log-hs-CRP remained to be positively associated, while serum albumin level and GFR negatively associated with A-FABP level.

Our study had some limitations. First, this study had a cross-sectional design without a control group with a limited number of participants enrolled and the possibility of bias cannot be excluded. Second, pharmacological interventions have been shown to influence serum A-FABP in humans. Thiazolidinedione increases plasma A-FABP concentrations in type 2 DM subjects [23]. Another study noted that canagliflozin increases serum A-FABP levels in diabetic patients [32]. Our patients did not use any the sodium/glucose cotransporter 2 (SGLT2) inhibitor. Our results did not show a relationship between thiazolidinedione, stains, fibrates or other antidiabetic drugs, and serum A-FABP in the diabetic patients studied. Further studies are required to elucidate the relationship between medication and A-FABP in type 2 DM patients.

## 5. Conclusion

In summary, the present study showed that serum A-FABP level was positively associated with MetS in the type 2 DM patients. In addition, body fat mass, log-creatinine, female DM patients, and log-hs-CRP are positively correlated, while serum albumin and GFR are negatively correlated with A-FABP level.

## Figures and Tables

**Table 1:** Clinical variables of the 165 diabetic patients with or without metabolic syndrome.

Items	All participants (*n* = 165)	No metabolic syndrome (*n* = 52)	Metabolic syndrome (*n* = 113)	*P* value
Age (years)	65.00 (57.00–70.00)	62.00 (56.25–67.00)	66.00 (57.50–71.00)	0.159
Height (cm)	161.75 ± 8.41	163.20 ± 7.86	161.08 ± 8.61	0.132
Body weight (kg)	70.69 ± 13.36	63.09 ± 8.88	74.19 ± 13.65	<0.001^*∗*^
Body mass index (kg/m^2^)	26.91 ± 3.94	23.62 ± 2.30	28.43 ± 3.60	<0.001^*∗*^
Body fat mass (%)	31.49 ± 7.55	23.36 ± 5.89	34.31 ± 6.50	<0.001^*∗*^
Waist circumference (cm)	90.69 ± 9.27	82.84 ± 7.16	94.30 ± 7.79	<0.001^*∗*^
Systolic blood pressure (mmHg)	142.470 ± 19.77	129.83 ± 14.75	148.28 ± 19.10	<0.001^*∗*^
Diastolic blood pressure (mmHg)	82.77 ± 11.07	76.85 ± 9.02	80.50 ± 10.89	<0.001^*∗*^
Albumin (mg/dL)	4.28 ± 0.27	4.27 ± 0.20	4.29 ± 0.29	0.565
Total cholesterol (mg/dL)	162.25 ± 30.69	161.962 ± 29.54	162.39 ± 31.33	0.934
Triglyceride (mg/dL)	115.00 (85.00–172.00)	92.50 (61.25–125.75)	127.00 (93.50–192.00)	<0.001^*∗*^
HDL-C (mg/dL)	46.69 ± 12.58	51.92 ± 14.47	44.28 ± 10.86	<0.001^*∗*^
LDL-C (mg/dL)	99.92 ± 26.58	97.40 ± 23.85	101.08 ± 27.77	0.411
Fasting glucose (mg/dL)	138.00 (121.00–175.00)	125.50 (116.250–157.75)	143.00 (126.00–179.50)	0.033^*∗*^
Glycated hemoglobin (%)	7.50 (6.60–9.00)	7.00 (6.25–8.10)	7.90 (6.70–9.30)	0.007^*∗*^
Blood urea nitrogen (mg/dL)	16.00 (12.00–18.50)	15.00 (12.00–17.00)	16.00 (13.005–19.50)	0.020^*∗*^
Creatinine (mg/dL)	0.90 (0.70–1.00)	0.90 (0.70–1.00)	0.80 (0.70–1.00)	0.741
Glomerular filtration rate (mL/min)	87.23 ± 27.80	74.70 ± 21.15	65.96 ± 20.31	0.021^*∗*^
hs-CRP (mg/dL)	0.08 (0.05–0.25)	0.05 (0.05–0.11)	0.11 (0.05-0.34)	<0.001
Insulin (*μ*IU/mL)	6.93 (3.62–14.64)	3.74 (2.10–6.10)	9.63 (5.27–17.68)	<0.001^*∗*^
HOMA-IR	2.47 (1.24–5.08)	1.26 (0.77–2.14)	3.55 (1.91–6.93)	<0.001^*∗*^
A-FABP (ng/mL)	28.49 ± 15.55	20.05 ± 9.91	32.37 ± 16.16	<0.001^*∗*^
Female (%)	73 (44.2)	14 (26.9)	59 (52.2)	0.002^*∗*^
Hypertension (%)	86 (52.1)	17 (32.7)	69 (61.1)	0.001^*∗*^
Smoking (%)	17 (10.3)	6 (11.5)	11 (9.7)	0.723

Values for continuous variables are given as means ± standard deviation and are tested by Student's *t*-test; variables not normally distributed are given as medians and interquartile range and are tested by Mann–Whitney *U* test; values are presented as number (%) and analysis was done using the chi-square test.

HDL-C, high-density lipoprotein cholesterol; LDL-C, low-density lipoprotein cholesterol; hs-CRP, high-sensitivity C-reactive protein; HOMA-IR, homeostasis model assessment of insulin resistance; A-FABP, adipocyte fatty acid binding protein.

^*∗*^ *P* < 0.05 was considered statistically significant after Student's *t*-test or Mann–Whitney *U* test.

**Table 2:** Clinical characteristics and fasting serum adipocyte fatty acid binding protein levels of 165 diabetic patients.

Characteristic		Number (%)	A-FABP (ng/mL)	*P* value
Gender	Male	92 (55.7)	23.90 ± 12.44	<0.001^*∗*^
	Female	73 (44.3)	34.27 ± 17.16	
Hypertension	No	79 (47.9)	26.16 ± 15.49	0.065
	Yes	86 (52.1)	30.63 ± 15.38	
Smoking	No	148 (89.7)	28.54 ± 15.55	0.907
	Yes	17 (10.3)	28.07 ± 16.03	
Statin	No	84 (50.9)	26.83 ± 13.07	0.164
	Yes	81 (49.1)	30.21 ± 17.68	
Fibrate	No	156 (94.5)	28.14 ± 15.73	0.229
	Yes	9 (5.5)	34.57 ± 10.78	
Metformin	No	74 (44.8)	28.47 ± 16.54	0.986
	Yes	91 (55.2)	28.51 ± 14.79	
Sulfonylureas	No	77 (46.7)	29.07 ± 16.50	0.654
	Yes	88 (53.3)	27.98 ± 14.75	
DDP-4 inhibitor	No	65 (39.4)	28.03 ± 17.09	0.763
	Yes	100 (60.6)	28.79 ± 14.54	
Thiazolidinediones	No	160 (97.0)	28.26 ± 14.60	0.282
	Yes	5 (3.0)	35.88 ± 37.05	
Insulin	No	122 (73.9)	28.39 ± 15.68	0.887
	Yes	43 (26.1)	28.78 ± 15.34	

Data are expressed as means ± standard deviations.

^*∗*^ *P* < 0.05 was considered statistically significant after Student's *t*-test.

DDP-4, dipeptidyl peptidase 4.

**Table 3:** Correlation of fasting serum adipocyte fatty acid binding protein levels and clinical variables by univariable linear regression analyses among the 165 diabetic patients.

Items	Beta	*P* value
Log-age (years)	0.115	0.140
Height (cm)	−0.293	<0.001^*∗*^
Body weight (kg)	−0.031	0.696
Body mass index (kg/m^2^)	0.173	0.026^*∗*^
Body fat mass (%)	0.394	<0.001^*∗*^
Waist circumference (cm)	0.166	0.033^*∗*^
Systolic blood pressure (mmHg)	0.324	<0.001^*∗*^
Diastolic blood pressure (mmHg)	0.173	0.026^*∗*^
Albumin (mg/dL)	−0.259	0.001^*∗*^
Total cholesterol (mg/dL)	0.179	0.022^*∗*^
Log-triglyceride (mg/dL)	0.227	0.003^*∗*^
HDL-C (mg/dL)	−0.041	0.600
LDL-C (mg/dL)	0.099	0.204
Log-glucose (mg/dL)	0.133	0.088
Log-HbA1c (%)	0.225	0.004^*∗*^
Log-BUN (mg/dL)	0.363	<0.001^*∗*^
Log-creatinine (mg/dL)	0.257	0.001^*∗*^
Glomerular filtration rate (mL/min)	−0.343	<0.001^*∗*^
Log-hs-CRP (mg/dL)	0.212	0.006^*∗*^
Log-insulin (*μ*IU/mL)	0.214	0.006^*∗*^
Log-HOMA-IR	0.218	0.005^*∗*^

Data of age, triglyceride, glucose, HbA1c, BUN, creatinine, and hs-CRP levels showed skewed distribution and therefore were log-transformed before analysis.

^*∗*^ *P* < 0.05 was considered statistically significant after univariable linear analyses.

Abbreviations: HDL-cholesterol, high-density lipoprotein cholesterol; LDL-cholesterol, low-density lipoprotein cholesterol; BUN, blood urea nitrogen; hs-CRP, high-sensitivity C-reactive protein; HOMA-IR, homeostasis model assessment of insulin resistance.

**Table 4:** Multivariable stepwise linear regression analysis of gender, height, body mass index, waist circumference, body fat mass, systolic blood pressure, diastolic blood pressure, albumin, total cholesterol, log-triglyceride, log-HbA1c, log-BUN, log-creatinine, glomerular filtration rate, log-CRP, log-insulin, and log-HOMA-IR: correlation to fasting serum adipocyte fatty acid binding protein level among 165 diabetic patients.

Items	Beta	Adjusted *R* ^2^	Adjusted *R* ^2^ change	*P* value
Body fat mass (%)	0.211	0.150	0.150	<0.001^*∗*^
Log-creatinine (mg/dL)	0.843	0.290	0.140	<0.001^*∗*^
Female	0.513	0.341	0.051	<0.001^*∗*^
Albumin (mg/dL)	−0.180	0.370	0.029	0.004^*∗*^
Log-hs-CRP (mg/dL)	0.154	0.390	0.020	0.013^*∗*^
Glomerular filtration rate (mL/min)	−0.366	0.402	0.012	0.043^*∗*^

^*∗*^ *P* < 0.05 was considered statistically significant after multivariable stepwise linear regression analyses.

hs-CRP, high-sensitivity C-reactive protein; HOMA-IR, homeostasis model assessment of insulin resistance.
